# Access to effective healthcare: effective self-management support intervention for patients with a chronic condition and a low social economic status: a systematic review

**DOI:** 10.1186/1472-6955-14-S1-S7

**Published:** 2015-10-08

**Authors:** Ann Van Hecke, Maud Heinen, Paz Fernandez-Ortega, Marit Graue, Jeroen Hendriks, Bente Høy, Sascha Köpke, Maria Lithner, Betsie van Gaal

**Affiliations:** 1 1University centre for Nursing and Midwifery, Ghent University, De Pintelaan 185, Ghent, Belgium; 2 Radboudumc, Radboud Institute for Health Sciences, IQ healthcare, Nijmegen, the Netherlands; 3 Institut Català d'Oncologia. Hospital Duran i Reynals / Nursing School, Department of Public Health, Mental Health and maternoinfantil, Barcelona University UB, Barcelona, Spain; 4 Faculty of Health and Social Sciences, Centre for Evidence-Based Practice, Bergen University College, Bergen, Norway / Department of Paediatrics, Haukeland University Hospital, Bergen, Norway; 5 Centre for Heart Rhythm Disorders, Royal Adelaide Hospital and University of Adelaide, South Australian Health and Medical Research Institute, Adelaide, Australia / Department of Medical and Health Sciences, Linköping University, Linköping, Sweden; 6 Department of Health care and Social Sciences, VIA University College Hedeager 2, Aarhus N, Denmark; 7 Institute for Social Medicine and Epidemiology, Nursing Research Unit, University of Lübeck, Germany; 8 Department of Surgery, Skane University Hospital Lund, Sweden

## Background

Access to effective healthcare is in particular challenging for vulnerable and socially disadvantaged patients. Patients with chronic conditions are over-represented in these lower socioeconomic (LSES) groups. No generic review integrating the evidence on Self-Management support interventions in LSES patients with different chronic conditions exists.

## Aims

To provide an overview of the effectiveness concerning patient-relevant outcomes of Self-Management support interventions and their components in chronically ill patients with a LSES.

## Methods

Six databases were explored from 2000 up to December 2013. 3195 abstracts were screened. 27 studies were included. Data extraction and quality assessment were performed by five pairs of independent reviewers.

## Results

The main focus was on SMSI in patients with diabetes, in the USA and 2 studies were conducted in Europe. 14 studies explicitly cited an underlying theoretical basis for the SMSI. The most frequently used Self-Management support interventions components were: lifestyle advice (n=25), information provision (n=24), and symptom management (n=22). Problem solving (n=13) and goal setting (n=13) were frequently integrated in the Self-Management support interventions. In 11 studies the Self-Management support interventions was specifically adapted to the needs of LSES patients. No differences in positive outcomes were found when stratified for theory-based Self-Management support interventions or for number of patients included. 

## Conclusions

Very few RCTs on Self-Management support interventions were performed in Europe. Limited evidence exists for the effectiveness of Self-Management support interventions in LSES patients and its linkages with components and strategies of these interventions. Therefore, to improve access to healthcare for all patients, it is important to develop and evaluate effective SMSI for these vulnerable patients.

